# PPARs in Irradiation-Induced Gastrointestinal Toxicity

**DOI:** 10.1155/2010/528327

**Published:** 2009-11-22

**Authors:** Christine Linard, Maâmar Souidi

**Affiliations:** Institute for Radioprotection and Nuclear Safety, B.P. no.17, 92262 Fontenay aux Roses Cedex, France

## Abstract

The use of radiation therapy to treat cancer inevitably involves exposure of normal tissues. Although the benefits of this treatment are well established, many patients experience distressing complications due to injury to normal tissue. These side effects are related to inflammatory processes, and they decrease therapeutic benefit by increasing the overall treatment time. Emerging evidence indicates that PPARs and their ligands are important in the modulation of immune and inflammatory reactions. This paper discusses the effects of abdominal irradiation on PPARs, their role and functions in irradiation toxicity, and the possibility of using their ligands for radioprotection.

## 1. Introduction

Recent advances in radiotherapy delivery, such as the development of dose-sculpting techniques, have led to an overall reduction in normal tissue exposure during radiation therapy. Nevertheless, radiation toxicity to normal tissue remains the most important dose-limiting factor in radiotherapy and is a major obstacle to uncomplicated cancer cure. Gastrointestinal symptoms after pelvic radiotherapy affect the patient's quality of life, are substantially more common than generally recognized, and are frequently inadequately managed [1, 2]. They develop because radiation induces changes in one or more specific physiological functions in widely separated parts of the gastrointestinal tract that lie in the path of the radiotherapy beam.

 Among the potential molecular targets for the treatment or even prevention of these disturbances are peroxisome proliferator-activated receptors (PPARs), a subclass of the nuclear hormone receptor superfamily. The two PPAR isotypes covered in this article have been identified in vertebrates: PPAR-*α*, and PPAR-*γ*. The PPAR-*α* isoform is abundantly expressed in the liver and is expressed as well in the kidney, testes, heart, small intestines, pancreas and smooth muscle. It is also detectable in the lungs, placenta, and adipose tissue. PPAR-*γ* is specifically expressed at high levels in the adipose tissue but is also detected in the intestines, particularly the colon [3]. Relatively low PPAR mRNA levels are observed in the skeletal muscle, liver, and bone marrow stromal cells. In view of its expression and involvement in immune response and cell proliferation, PPAR-*γ* has become an especially important research topic in gastroenterology, particularly in two important disorders—inflammatory bowel diseases (IBDs) and colon cancer. At this time, relatively little is known about the potential protective and healing characteristics of PPAR-*γ* in radiotherapy-induced bowel damage.

## 2. Molecular Mechanisms of PPAR Activity

### 2.1. Mechanism of Transcriptional Transactivation

The variety of their functions makes it difficult to define the molecular mechanisms of PPAR activity. PPAR-*γ* acts by blocking gene transcription by transrepression, a feature of transcriptional crosstalk between nuclear receptors (NRs) and signaling cascades that modulates inflammation and immunity in a variety of cells, including the epithelial cells of the gut. Several models have been suggested to describe transrepression by PPAR-*γ* interacting with the transcription factor nuclear factor *κ*B (NF-*κ*B). NF-*κ*B plays an important role in the regulation of immune and inflammatory responses, for it induces the expression of diverse target genes that promote cell proliferation, regulate apoptosis, facilitate angiogenesis and, in carcinogenesis, stimulate invasion and metastasis [4].

 In one coactivator competition model, NF-*κ*B and PPARs use an overlapping set of coactivator proteins [5] and compete with each other to bind to these coactivators. For example, under steady-state conditions, some genes (e.g., iNOS) are occupied and actively repressed by the nuclear receptor corepressor (NCoR) that serves simultaneously to repress PPAR-mediated transcription. The switch from repression to activation requires a reduction in affinity for the corepressor. In addition to the conformational change in the ligand binding domain, ligand binding removes NCoR complexes from promoters of nuclear receptor target genes and thus increases affinity for coactivators [6]. The subsequent finding that transrepression still occurs in the presence of excess coactivators [7] raises serious questions about this model, however.

 A second proposed model involves direct interactions between nuclear receptors and negatively regulated transcription factors, interactions that result in the inhibition of the DNA-binding or transactivating activity of one or both factors [5, 8]. In endothelial cell lines, for example, PPAR-*α* activation inhibits the inflammatory response by direct protein-protein interaction with NF-*κ*Bp65 [9]. Similarly, PPAR-*γ* inhibits the production of cytokines in LPS-stimulated macrophages by direct interaction with NF-*κ*Bp65/p50 [10]. In smooth muscle cells and hepatocytes, PPAR ligands induce the expression of I*κ*B*α*, which leads to retention of the NF-*κ*B subunits in the cytoplasm and the consequent suppression of their DNA binding activity [11]. It is worth pointing out that PPAR-*γ* ligands, besides promoting PPAR-*γ* interaction with NF-*κ*B subunits, may also act independently of PPAR-*γ*. For example, the PPAR-*γ* ligand 15d-PGJ_2_ inhibits the secretion of TNF-*α* and IL-6 in macrophages stimulated by LPS and directly blocks activity of the I*κ*B kinase complex, completely independently of PPAR-*γ* [12].

 More recent studies have produced another model involving a corepressor-dependent mechanism. Ligand-binding of PPAR-*γ* induces the SUMOylation of a fraction of some PPAR-*γ* molecules, which bind to NCoR and prevent its clearance from the promoter. This leads to a sustained repressed state. Using yeast two-hybrid screen assays, Pascual et al. [13] showed that PPAR-*γ* interacts with the protein inhibitor of the activated transcription factor, STAT-1 (PIAS1). The physiological role of PIAS1 is to facilitate the localization of PPAR-*γ* to the NCoR complexes on the promoter of inflammatory genes, including iNOS, in the presence of PPAR-*γ* ligands. Consequently, NF-*κ*B-mediated inflammatory gene expression is downregulated [14].

### 2.2. Natural and Synthetic Ligands

PPAR-*γ* receptors are activated by several natural lipophilic ligands, including long-chain polyunsaturated fatty acids, arachidonic acid metabolites derived from the cyclooxygenase, and lipoxygenase pathways (such as 15-deoxy-d-12,14-prostaglandin J2 (15PG-J2) and 15-HETE) and fatty acid-derived components of oxidized low density lipoproteins (OxLDLs) (such as 9-hydroxyoctadecadienoic acid (9-HODE) and 13-hydroxyoctadecadienoic acid (13-HODE) [15]. The antidiabetic class of thiazolidinedione drugs includes troglitazone, rosiglitazone, pioglitazone and ciglitazone, all synthetic ligands of PPAR-*γ*. Other synthetic compounds that can function as ligands include certain nonsteroidal anti-inflammatory drugs (NSAIDs), such as indomethacin, ibuprofen, flufenamic acid, and fenoprofen [16]. Saturated and unsaturated fatty acids are the primary natural PPAR-*α* ligands, and eicosanoids and leukotriene *β*4 are among the higher affinity endogenous PPAR-*α* ligands. The best-studied synthetic PPAR-*α* ligands are the fibrate class of hypolipidemic drugs (bezafibrate, clofibrate, and fenofibrate), which have been used therapeutically in humans for many years [16].

## 3. Pathophysiology of Normal Intestinal Tissue after Pelvic Irradiation

Any clinician involved in the treatment of pelvic malignancies with radiation is aware of the danger of colorectal injury. Although the severe effects were described long ago, consideration of lesser degrees of colorectal injury is much more recent [1, 2]. It remains difficult to define exactly when symptoms start to affect quality of life and why some people seek help for specific gastrointestinal symptoms while others do not. The pathological processes of radiation injury begin immediately after radiation exposure, but its clinical and histological features may not become apparent for weeks, months, or even years afterwards. Radiation injury is commonly classified as acute, consequential, or late effects, according to the time of appearance of symptoms. Acute (early) effects are observed during the second week of treatment (when histological changes probably reach their zenith) and peak by the fourth to fifth weeks (when the histological changes have stabilized or are improving). Some randomized studies show that the prevalence of mild acute intestinal effects ranges from 6 to 37% and severe effects (resulting in treatment interruption) from 0 to 10% [17]. Consequential effects appear later and are caused by persistent acute damage [18]. They tend to peak at 18 months [19]. These symptoms concern fewer patients (half of those with late effects) but are clinically important because of their chronic progressive nature and their significant long-term morbidity. Late effects emerge months to years after radiation exposure. Prevalence estimates of moderate and severe late bowel effects vary from 5% to 30% [20, 21] and affect patients' quality of life. Indeed 2.5% of all pelvic radiotherapy patients require surgery as a result of its complications.

 Among the most common side effects of pelvic radiotherapy are acute radiation-induced proctosigmoiditis, enteritis or colitis, depending on the symptoms and localization. They result from delivering radiation to the distal colon and rectum during radiotherapy that touches other pelvic structures. The acute symptoms are pain and diarrhea or constipation due to the loss of epithelial integrity and increased mucus secretion. While the specific causes of constipation have not been investigated, the many causes of diarrhea include accelerated small and large bowel transit, bacterial overgrowth, and malabsorption of bile salts. Consequently, the tissue develops edema, and hyperemia.

 The most common late effects include increased stool frequency and urgency, spotting of blood, and partial incontinence. Less common are ulceration, severe bleeding, pain, stricture, severe incontinence, and fistula [17]. Ulceration results from the tissue necrosis induced by the release of radical species, persistence of the inflammatory process, and the local ischemia associated with the vascular damage. Severe complications, such as bleeding, fistulas, and obstruction, may require surgical intervention. Ischemia and fibrosis in the submucosa and muscularis layer are mainly responsible for these effects, which are accompanied by vascular sclerosis, collagen deposition, and abnormal fibroblast activity. Compromised vascularization of fibrotic tissues [22] contributes to an adverse postoperative outcome, and symptoms of this radiation enteropathy frequently persist after surgery. Proctitis alone is estimated to account for more than 75% of all gastrointestinal radiation injuries [23] and usually begins in the second or third week of radiotherapy. Acute radiation-induced proctosigmoiditis is quite similar to distal ulcerative colitis, both in its symptoms and its acute histopathologic effects. Studies of patients receiving bladder and prostate radiotherapy demonstrate continued active inflammation and repair processes at 3 months after treatment [24]. The acute and chronic effects of radiation to the rectum appear to be correlated [25–27].

## 4. PPARS and Control of Gastrointestinal Inflammation

### 4.1. Inflammatory Response

Inflammation is a major component of early healing, and its control is essential for efficient repair. It plays a causative role in radiation-induced toxicity. The first observation of this inflammatory process showed that levels of eicosanoids, leukotriene B4, thromboxane B2, and prostaglandin E2 all rise markedly in the rectum in response to pelvic irradiation and fall after radiotherapy is complete [28]. Irradiation activates various cell signaling pathways that lead to expression and activation of proinflammatory and profibrotic cytokines [29–32], to vascular injury [33] and to activation of the coagulation cascade [34, 35].

 Intestinal homeostasis is generally maintained through balanced immune and inflammatory responses, epithelial integrity, and adequate lymphoepithelial interactions. Su et al. [36] did essential work on the role of PPAR-*γ* in intestinal inflammation, developing the first model of colitis induced by oral administration of dextran sodium sulphate [36, 37] and showing the response of inflammation to treatment by PPAR-*γ* ligands. Another model of colitis, induced by intrarectal administration of 2,4,6-trinitrobenzene sulfonic acid (TNBS), showed that treatment with troglitazone attenuates colitis [38]. Because it has been reproduced by activation of RXR with specific rexinoids, this beneficial effect has been directly attributed to the RXR/PPAR-*γ* heterodimer [38]. Further evidence in support of the involvement of this heterodimer comes from the enhanced susceptibility of PPAR-*γ* ^+/−^ and RXR^+/−^ mice to TNBS-induced colon inflammation [38]. The high expression of PPAR-*γ* in epithelial cells suggests that these cells are the main target of the RXR/PPAR-*γ* activators, and this hypothesis is reinforced by the persistence of inflammation in deeper colonic layers in animals treated with PPAR-*γ* or RXR agonists [38].

 The use of exogenously administered PPAR-*γ* ligands alone in this study left several questions open, especially since only disease-activity measurements were considered, rather than other objective colitis indicators. Can the anti-inflammatory action of PPAR-*γ* activation be proved by other objective indicators of colitis-associated inflammation? Does endogenous PPAR-*γ* play an anti-inflammatory role in physiological conditions? Are the levels of PPAR-*γ* protein lower in IBD patients than those in healthy controls and does that make these patients more susceptible to chronic inflammation? Nakajima et al. answered these questions, showing not only that PPAR-*γ* ligands inhibit inflammation, but also that a PPAR-*γ* deficiency is associated with aggravated injury [39]. This report showed clearly that PPAR-*γ* functions as an endogenous anti-inflammatory substance in the intestines and thus that a decrease in PPAR-*γ* levels may exacerbate inflammation. IBD patients may have low levels of PPAR-*γ* in their intestinal mucosa, potentially predisposing them to unrestrained inflammation, as observed in PPAR-*γ*-deficient mice. Human studies are rare but appear to indicate that patients with ulcerative colitis, but not those with Crohn's disease, have reduced levels of PPAR-*γ* protein in their colonic epithelial lining [40]. This discrepancy between the two diseases has not yet been explained.

 Study of the role of PPARs in irradiation-induced inflammatory process began only recently. Experiments in our laboratory have measured the level of colonic PPARs after abdominal irradiation. As in patients with ulcerative colitis, PPAR-*α*, -*γ*, and heterodimer RXR*α* expression (of both genes and proteins) fell drastically three days after a single 10-Gy abdominal irradiation, and acute intestinal injury was observed [41]. Similarly Zhao et al. showed that whole-body irradiation downregulated PPAR-*α* and -*γ* protein levels in the mouse kidney [42]. The PPARs, notably PPAR-*α* and RXR-*α*, have been shown to be highly radiosensitive, with PPAR repression observed from the first doses during a fractionated colorectal irradiation (4 Gy/fraction, 3 fractions/week and total dose 52 Gy) that mimicked a radiotherapy protocol in a rat model (unpublished results).

 In vivo*, *irradiation produces an acute and a chronic increase in ROS generation that leads to persistent chronic oxidative stress [43]. PPAR expression is sensitive to oxidative stress and to inflammatory processes, as shown by the decreased PPAR-*α* expression induced by LPS or proinflammatory cytokines such as tumor necrosis factor-*α* (TNF-*α*) [44]. In the acute phase of irradiation, the downregulation of PPARs may be a mechanism that contributes to the development or exacerbation of inflammation. Because PPAR-*γ*/RXR*α* is highly expressed in epithelial cells and given that irradiation may erode the epithelial layer, it was thought that PPAR downregulation might well be correlated with direct irradiation of the epithelium. But at the doses delivered and during the experimental period, no epithelial erosion was observed [41]. Currently, we have no explanation for the impairment of PPAR expression that is induced by irradiation. The direct impact of this impairment on the severity of radiation enteritis remains to be determined. Zhao et al. showed that the reduction of PPAR levels aggravates radiation nephropathy [42]. Their study shows that radiation-induced apoptosis is inhibited in PPAR-*α* knockout mice by the enhanced activation of NF-*κ*B and the increased expression of prosurvival factors. The activation of apoptosis signaling pathways prevents the DNA damage that increases the risk of genomic instability, mutations, and long-term carcinogenesis. The absence of this response in PPAR knockout mice might thus increase carcinogenesis in these mice.

 Many molecular mechanisms and cellular targets play a role in controlling colonic inflammation by PPAR expression and activation in epithelial and various types of immune cells, especially macrophages. Activation of PPAR-*γ* in the colon inhibits mucosal production of inflammatory cytokines (TNF-*α*, IL-1*β*, and IL-6) by downregulating the NF-*κ*B and MAP kinase signaling pathways [38]. Studies with cell culture models have established that PPAR-*γ* agonists such as thiazolidinedione can reduce NF-**κ**B activation and inflammatory gene expression in colonic epithelial cells [45], macrophages [46–48], dendritic cells [49], and T cells [50, 51]. Although the efficacy of thiazolidinedione depends on numerous factors including the cell model, concentration, duration, type of thiazolidinedione (rosiglitazone, pioglitazone) used, and inflammatory model, it is nonetheless generally accepted that these drugs can reduce inflammatory gene expression.

 In our laboratory we tested the strength of the reduction of the inflammatory process induced by GW1929, a nonthiazolidinedione that has been identified as a high-affinity ligand for human PPAR-*γ* and is reported to be more potent than troglitazone [52]. Surprisingly, the treatment exacerbated the irradiation-induced inflammatory process, causing drastic weight loss and more severe mucosal inflammation (unpublished results). These findings suggest that it is absolutely necessary to analyze inflammatory and immune status before treatment. No treatment is universal for every inflammatory disease of the colon. Questions remain about the role of inflammatory (macrophages, T cells) and epithelial cells in PPAR response in other models of acute and chronic enteritis, such as radiotherapy-induced inflammatory intestinal response. The diversity of colitis models may be helpful in demonstrating the importance of targeting the specific ligand-activated PPAR.

### 4.2. PPARs and Macrophages

It is well established that each PPAR isoform is expressed in monocyte/macrophage lineages and influences their phenotypes [46]. In monocytes/macrophages, PPAR-*γ* activation inhibits the expression of inflammatory cytokines such as TNF-*α*, IL-1*β*, and IL-6. Similarly, in macrophages, treatment by PPAR-*γ* ligands induces a resting phenotype and suppresses iNOS [48]. These observations indicate that PPAR-*γ* might be a target for anti-inflammatory therapy. More recently, studies with mutations that disrupt one of the PPAR genes confirm the in vivo anti-inflammatory effects of PPARs. Thus, Chawla et al. [53] reported that both 15d-PGJ_2_ and troglitazone exert anti-inflammatory effects in macrophages derived from mice homozygous for a null mutation in the PPAR-*γ* gene and therefore appear to work by a PPAR-*γ*-independent mechanism. In their study of PPAR-*γ*-deficient macrophages, Crosby et al. [54] showed that PPAR-*γ* is not necessary for the successful inhibition of inflammatory mediators, in particular, iNOS, by synthetic agonists.

 Recently, we showed that 5-aminosalicyclic acid (5-ASA) reduces the overexpression of TNF-*α*, MCP1, and iNOS that is induced during the acute phase of irradiation. These effects could be seen by the reduction of macrophage infiltration in the mucosa [41]. It thus appears to us that the role of PPAR-*γ* may be overestimated in these irradiation protocols, since the ligands are used at concentration exceeding those required to bind PPAR-*γ* [53]. 5-ASA acts on inflammatory processes even at the time when irradiation substantially impairs PPAR/RXR*α* expression. These observations show that the anti-inflammatory effects of PPAR-*γ* ligands in inflammatory cells may be independent of their ability to activate PPAR-*γ* and may involve instead mechanisms such as interference with early signals of the transduction cascade that block NF-*κ*B transcription (see Section 4.4).

 In the past several years, studies of macrophages have focused on their role as key cells of innate immune system and their fundamental activities in killing and removing foreign organisms and cell debris. Response to macrophage stimulation is usually seen as proinflammatory and proimmunogenic. More recently, different forms of macrophage activation have been proposed with noninflammatory and even anti-inflammatory consequences. A state of macrophage activation, in response to infectious agents, particularly Mycobacteria, involves IFN*γ* and other cytokines, in conjunction with stimulation by LPS or other Toll-like receptor (TLR) ligands. These so-called classically activated or M1 macrophages have upregulated iNOS and COX2. These cells play an important role in protection against intracellular pathogens and cancer cells.

 More recently, research has focused on the activation of macrophages that produce inflammatory mediators. General suppression is achieved after stimulation with IL-10, but exposure to IL-4 or related cytokines initiates a so-called “alternatively activated” or M2 macrophage [55]. Its capacity for an oxidative burst, that is, to produce inflammatory mediators, such as iNOS, in response to LPS, is reduced. These cells have distinct functional properties, and are integrated in polarized Th2 responses, tissue remodeling, and repair [55].

 Although sufficient data are not yet available, macrophage polarization is an interesting vantage point for studying the effects of irradiation and their long-term consequences. In a Danish clinical study during radiotherapy of the pelvic-abdominal area, immunohistochemical staining of the CD68 antigen showed increased density of macrophages at 2 weeks (during the radiotherapy), but no further change at 6 weeks (the end of the protocol). The stained macrophages were localized mainly beneath the epithelium in close proximity and intermingled with other inflammatory cells in the lamina propria [56]. In our laboratory fractionated irradiation of an animal colorectal model confirmed and extended these observations; macrophage immunostaining increased over the period of the protocol, while 6 months after the protocol intended, staining was diminished compared with the control animals [57].

 In vivo, irradiation modulates the functional abilities of some cells, in particular, NO production by activated macrophages in an arthritis model [58]. Activation of macrophages to produce iNOS depends on the dose delivered; a dose-dependent modulation of the NO pathway was observed with radiation doses used in anti-inflammatory radiotherapy and with superstimulation by the high radiation doses used for cancer treatment. PPAR-*γ* expression is not necessary for some macrophage functions, including activation and differentiation of this cell lineage. PPAR-*α* is thought to be involved in NO pathways, including in the inhibition of iNOS expression by murine macrophages [59]. Inversely, PPAR downregulation is associated with the overexpression of macrophage-related inflammatory mediators, including iNOS, TNF-*α*, and MCP1, and with macrophage infiltration [41] during the acute phase of irradiation. Interestingly, 6 months after the end of the radiotherapy protocol in the rodent, the PPAR level returned to normal but iNOS expression was cut in half. A potential explanation of this finding is that the modification of macrophage polarization is a long-term effect induced by irradiation. One study shows that peritoneal resident macrophages induced by the typical M2 inducers, IL-4, strong inducers PPAR-*γ* (gene and protein) and thus suggests that high PPAR-*γ* activity is preferentially associated with M2 [60]. Overall, it is clear that the regulation of PPARs by pro- or anti-inflammatory signals is an important factor that triggers macrophage polarization. The functional activity of irradiation-induced macrophages and their involvement in long-term effects (fibrosis, lack of response to infection) must be considered in this light.

### 4.3. Immune Response to Irradiation: PPAR Involvement

Cytokines are especially important for regulating immune and inflammatory responses with pro- and anti-inflammatory functions, and perform crucial functions in controlling both the innate and adaptive immune response. Not only do cytokines govern the development and homeostasis of lymphocytes, but they also direct the differentiation of helper T cells and promote the generation of memory cells [61]. Immune-mediated damage to intestinal mucosal surfaces can be triggered by both polarized Th1 and Th2 effector CD4^+^ T cells, and it can be improved or prevented. Three functional categories of terminally differentiated immune cells such as T helper (Th) cells have been characterized on the basis of their cytokine production and homing capacity: Th1 (IFN-*γ*, TNF-*α*, IL-12), Th2 (IL-4, IL13), and regulatory Th cells (Treg) that produce IL-10 able to inactivate Th1 and Th2. Th1 cells are mainly involved in cell-mediated immune responses; whereas Th2 cells participate in humoral responses and promote growth and differentiation of mast cells and eosinophils. Because the Th1 and Th2 subpopulations tend to function antagonistically towards one another, the persistence of disease susceptibility and resistance depends on the cytokine profile secreted by each type. One molecular mechanism for cell polarization may be the regulation of cytokine genes by transcription factors, although the expression of these genes, such as that for IFN-*γ*, can also be regulated at posttranscriptional stages. Induction of T-bet transcription factor expression in primary T cells is strongly correlated with Th1 lineage differentiation and IFN-*γ* expression. In Th2 lineage differentiation and expression of Th2 cytokines (IL-4, IL-5, and IL-13), on the other hand, the GATA3 transcription factor plays a role analogous to that of T-bet in Type 1 lineage differentiation and IFN-*γ* expression. GATA3 expression polarizes T cells towards a Th2 lineage and represses IFN-*γ* expression. It has been suggested that the balance between T-bet and GATA3 expression is crucial for Th1/Th2 T-cell differentiation [62]. Recent reports show that ionizing radiation induces the preferential differentiation of Th cells into Th2 cells in the spleen [63, 64] and more recently, in the intestines [65] where this Th2 dominance is characterized by repression of Th1-specific sets (IFN-*γ*, T-Bet). Indeed, irradiation initiates and maintains a Th2-like immune response over the long term [57]. Th2 cells play a critical role in the pathogenesis of radiation-induced pneumonitis, which precedes lung fibrosis [66]. PPAR-*γ* involvement has previously been described in the regulation of innate immune response [48], and more recently its role has been investigated in adaptive immunity [67, 68]. Although PPAR-*γ* is expressed on dendritic, T and B cells, little is known about their specific expression of PPARs, notably in colitis. PPAR-*γ* ligands have a negative effect on the APC functions of dendritic cells [69]. Remarkably, adoptive transfer of antigen-presenting dendritic cells pretreated with PPAR-*γ* resulted in CD4^+^ T cell anergy [70], characterized by impaired differentiation that led to the absence of both Th1 and Th2 cytokine production.

 PPAR-*α* and PPAR-*γ* are differentially expressed after lymphocyte activation. PPAR-*α* expression was downregulated after T cell activation while PPAR-*γ* expression increased under the same activating conditions. PPAR ligands inhibit the proliferation of T lymphocytes as well as of B cells [71] and repress the production of IFN-*γ*, TNF-*α*, and IL-2 [67]. Saubermann et al. [72] showed that the reduction in inflammation induced by dextran sodium sulphate, which has been noted with PPAR-*γ* ligand treatment, is associated with decreased IFN-*γ* and TNF-*α* and increased IL-4 and IL-10 expression. Consistent with this shift towards Th2 cytokine dominance, treatment by PPAR-*γ* ligands (troglitazone, pioglitazone, and rosiglitazone) increases the expression of the specific Th2 transcription factor, GATA-3. On the other hand, Jones et al. showed that CD4^+^ T cells lacking PPAR-*α* produce high levels of IFN-*γ*. These results indicate that the protective effects exhibited by PPAR-*γ* ligands in intestinal inflammation may be due to immune deviation away from Th1 and towards Th2 cytokine production. Surprisingly, by using another PPAR-*γ* ligand, 5-aminosalicylate, we restored the PPAR/RXR-*α* level and normalized the downregulation of the irradiation-induced IFN-*γ*/STAT1 pathway [57]. These results are inconsistent with reports that PPARs negatively regulate IFN-*γ* and presents an essential question: how may PPAR ligands modulate the overexpression of IFN-*γ* in a Th1 model of colitis and upregulate IFN-*γ* in Th2 colitis?

 The twofold immunological effect of PPAR ligands has been observed in two different acute lung inflammatory models—carrageenan-induced pleurisy [73] and bleomycin-induced lung injury [74]. In both, PPAR-*γ* ligands reduced inflammatory cell infiltration (by neutrophils and possibly other leukocytes). Others have hypothesized that PPAR-*γ* ligands act by a direct effect on the migration of inflammatory cells in response to endogenous chemoattractant [75]. Other studies show that PPAR-*γ* ligands may modulate leukocyte-endothelial interactions during inflammation through the regulation of endothelial adhesion molecules [74]. Considerable evidence suggests that PPAR activation may limit the endothelial responses that promote T cell adhesion and entry into the vessel wall.

 PPAR ligands may act during several different stages of acute irradiation effects. Firstly, PPARs may inhibit Th2 trafficking into the intestinal mucosa by interfering with the endothelial adhesion molecules. Secondly, in vivo irradiation leads to acute and chronic increases in ROS generation and thus to persistent oxidative stress [43]. The free radicals produced by irradiation cause vital cell damage that results in the apoptosis of cells in the first division or within the first few divisions. Some infiltrating cells, especially macrophages, serve as the major source of inflammatory molecules and can increase cell damage. Because Th1 cells are highly sensitive to apoptosis [76], modification of their oxidative status by PPARs may help to normalize the Th1/Th2 balance. Thirdly, we can rule out any direct action by PPAR ligands on transcription factors. Although no synthetic inhibitors of GATA-3 currently exist, PPAR-*γ* agonists have been shown to inhibit GATA-3 expression and Th2-driven inflammatory responses in murine models [77]. The yin-yang cytokine balance of the Th1 and Th2 cytokines is a common feature of inflammatory response and is thought to reflect the operation of a feedback control mechanism. The effects of PPARs on T lymphocytes are thus complex and require further study, especially in the field of radiopathology. Possible explanations for these observed differences include PPAR-independent effects of specific ligands or differences in the model system used.

### 4.4. Possible Mechanisms: Crosstalk with Signal Transduction Pathways

The mechanisms that perpetuate responses after irradiation remain a mystery. No evidence of the expression of immune cytokines (e.g., IL-2, IL-4, and IL-12) has been found in irradiated mouse brains [78], but immune mechanisms may have more importance in other tissues, such as the lung and the intestine [65, 79]. Mitotic death of specific cell populations responding to cell loss may result in lethal damage at different times after irradiation and “waves” of responses. Other possible mechanisms include dysregulated intercellular signaling due to the loss of specific cell populations, the disruption of negative regulatory cytokine pathways [80], or the development of hypoxia [81].

 It is thought that PPAR-*α* and PPAR-*γ* repress inflammation by physical interaction and form inactive complexes with the proinflammatory transcription factors NF-*κ*B and Activating Protein1 (AP1) [82]. These transcription factors normally induce the transcription of proinflammatory genes, such as cytokines (IL-6, TNF-*α*), iNOS [83] and COX-2 [84], chemokines (IL-8, MCP-1), and cellular adhesion molecules (VCAM1, ICAM1). It has also been suggested that PPAR-*γ* can interact with two transcription factors, signal transducer and activator of transcription (STAT) and nuclear factor of activated T-cells (NFAT), and thereby reduce the transcription of proinflammatory genes [82]. Another way in which PPAR-*α* and PPAR-*γ* control inflammation is more indirect, by influencing the transcription of genes that inhibit NF*κ*B signaling, such as I*κ*B*α* and IKK. For example, ligand-activated PPAR-*α* can induce expression of I*κ*B*α* [9]. By linking to NF*κ*B subunits, it prevents the translocation of NF*κ*B to the cell nucleus and the subsequent transcription of proinflammatory genes. The induction of I*κ*B*α* via PPAR-*α* is therefore positively correlated with decreased inflammation and thus helpful in preventing or countering low-level chronic inflammation. In the acute irradiation intestinal model, the PPAR ligand 5-ASA also acted by overexpressing I*κ*B*α* and thus reducing nuclear translocation and activation [41].

 Several reports indicate that activated PPAR-*γ* crosstalks with cytokine-mediated signal transduction pathways in the modulation of immune response. Several lines of evidence indicate that STAT signaling may be involved in the anti-inflammatory action of PPAR ligands [85]. Wang et al. explored the interactions between PPAR-*γ* and STAT3 in detail [86]. Two distinct PPAR-*γ* ligands suppress IL-6 activated STAT3 through various types of crosstalk including direct or mediated by the corepressor SMRT (silencing mediator of retinoid and thyroid hormone receptor). The exact mechanism through which PPAR-*γ* represses STAT3 is not yet fully elucidated. Most studies of multiple myeloma cells show that the inhibitory effects of the PPAR-*γ* ligand on STAT3 activity depend directly on the expression and activation of PPAR-*γ* [86]. A direct physical protein-protein interaction occurs between nuclear receptor PPAR-*γ* and activated transcription factor STAT3. Moreover, the enhancement of this interaction by 15-d-PGJ2, but not troglitazone, suggests that these two classes of PPAR-*γ* ligands inactivate STAT3 through different mechanisms.

 Interestingly in the intestinal irradiation model with PPAR-*α* and PPAE-*γ* repressed (gene and protein levels), STAT3 repression was also observed, again of both gene and protein levels [41]. By normalizing PPAR expression, 5-ASA elevated STAT3 protein levels (nucleus and cytosol). In addition the direct effect of PPAR-*γ* on STAT3 repression, an alternative mechanism for PPAR ligand-mediated STAT3 expression, has been suggested, in which the PPAR ligand acts indirectly by stabilizing inflammatory transcription factor suppressor molecules [87].

 Overwhelming evidence shows that STAT3 is the only obligate factor required for IL-10-mediated anti-inflammatory signaling [88]. In the mouse, thiazolidinedione treatment of acute colitis is accompanied by a marked increase in IL-10 expression [71], especially in mature dendritic cells and in activated CD4^+^ T cells; this increase depends on both dose and PPAR-*γ* [49] when the presence of a functional PPAR response element (PPRE) in the IL-10 promoter region has been demonstrated. As in colitis, intestinal irradiation represses IL-10 [89] and all strategies that interfere with irradiation-induced NF-*κ*B activation restore the IL-10 level, including 5-ASA treatment (unpublished results).

 The obligate role of STAT3 in IL-10 signaling raises the issue of pathway redundancy and specificity, for many receptors use STAT3. For example, IL-6 signaling also activates the STAT3 pathway but is incapable of activating the anti-inflammatory response. One explanation involves the effects of suppressor of cytokine signaling 3 (SOCS3) on the IL-6 receptor. SOCS3 plays an essential role as a negative inhibitor of IL-6 by interfering with STAT3 [90]. Overexpression of SOCS3 is observed immediately (3 hours) after intestinal irradiation [89] and the SOCS3 level remains high for a long time after irradiation ends (6 months) [57]. In our laboratory, we showed that interference with the NF-*κ*B pathway—either direct, by a specific inhibitor (CAPE), or indirect, by PPAR activation (5-ASA)—normalizes the SOCS3 level and in a roundabout way re-establishes the STAT3 level [41, 65]. STAT3 phosphorylation continues for a prolonged period in the absence of SOCS3 [90]. PPAR ligands can activate anti-inflammatory pathways both dependently and independently of IL-10, under the control of the negative regulatory influence of SOCS3. The multiplicity of crosstalk between nuclear receptors and transcriptional factors is an important factor that contributes to signal diversification and specification.

## 5. Radiotoxicity Prevention and Treatment

The improvement in survival after the development of new schedules and techniques for delivering radiotherapy underlines the importance of addressing the problems faced by long-term survivors. Priority must be given to assessing simple methods of preventing bowel toxicity in the first place, without compromising either tumor control or prevention of a secondary cancer. An earlier review of prophylactic treatments for radiation toxicity failed to find that any current regimen was effective [91].

 In the late 1970s, studies showed that 5-aminosalicylate is the active moiety of sulfasalazine in patients with ulcerative colitis and Crohn's disease. Since then, this agent, its efficacy, drug profile, precise indications, and adverse events have remained topics of unceasing discussion [92]. At the same time, controversy arose about the prevention of radiation-induced bowel toxicity. A small double-blinded, balanced and randomised trial study showed that acetylsalicylate was effective against the side effects of uterine radiotherapy; it reduced the number of bowel motions and relieved abdominal pain [93].

 More recently, Jahraus et al. conducted a double-blinded, randomized, placebo-controlled trial of balsalazide and observed a marked reduction in the classic symptoms of acute radiation-induced proctosigmoiditis [94]. Balsalazide is one of a class of functional drugs whose active metabolite is 5-ASA. It inhibits the synthesis and release of proinflammatory mediators (NO, leukotrienes) and the function of natural killer cells, mast cells, neutrophils, mucosal lymphocytes and macrophages [95]. These results were somewhat surprising, since several other large randomized studies show that 5-ASA either does not improve or worsens the symptoms of acute radiation enteritis. Early randomized trials of olsalazine [96] and mesalamine [97] were disappointing, with mesalamine showing no benefit and olsalazine showing an increased incidence of diarrhea. The negative result for olsalazine was not surprising, because a similar increase in diarrhea prevalence was seen in IBD patients [98]. A study of balsalazide found that it did not significantly alleviate clinical symptoms [99]. In contrast, the randomized, double-blind controlled trial of sulfasalazine conducted by Kiliç et al. in patients with a variety of pelvic radiotherapy malignancies reached the opposite conclusion. This larger, controlled study with a detailed grading of acute radiation enteritis reported that none of the patients in the sulfasalazine-treated group experienced Grade 4 diarrhea compared with the placebo group [100].

 The use of 5-ASA in radiation-induced gastrointestinal complications has long been the topic of debate. It would be more effective if given before the radiotherapy insult initiated the cascade of oxidative enzymes rather than after this cascade has already begun [96]. At this time, these inconsistent early clinical observations warrant further investigation into the immunobiology of PPARs and their potential role as a therapeutic target in the protection of patients undergoing radiotherapy. Overall, the benefit of 5-ASA appears to depend on its dose, formula, and mode of administration. The new-generation 5-ASA (i.e., Balsalazide), which produces a high concentration of active drug in the colon, seems off to a promising start in making pelvic radiotherapy more tolerable.

## 6. PPAR Ligand-Associated Toxicities

Wound healing after injury is a high priority for survival. In this situation, epithelial cells change their intracellular contacts, modify their matrix, proliferate, and migrate over the wound. Interestingly, each of these healing behavior is similarly involved in tumorigenesis and metastasis. Epithelia are highly susceptible to injury and also heal injuries effectively. At the same time, 95% of all cancer deaths are from epithelial tumors. Together these facts suggest that the repair mechanisms activated in response to injury may, if not controlled, promote cancer.

 Although PPARs may be involved in tumor-associated pathways, their regulation of wound-healing genes within specific tumors remains largely unexplored [101]. PPARs, especially PPAR-*γ*, are expressed or overexpressed in several abdominal malignancies, including colorectal carcinoma [102, 103], prostate carcinoma [104], and pancreatic carcinoma [105]. Another study reports that 8% of primary colorectal tumors harbor a loss of function mutation in one allele of the PPAR-*γ* gene and emphasizes the potential role of this receptor as a tumor suppressor in humans [106].

In vivo evidence to support an antitumorigenic role of PPAR-*γ* is also conflicting. Some experiments show that PPAR-*γ* can behave as a ligand-activated tumor suppressor. The ligands that activate PPAR-*γ* can inhibit proliferation and induce differentiation and apoptosis of a wide range of neoplastic cell types in vitro and in murine xenograft tumor models. PPAR*γ* ^−/−^** mice are more susceptible than wild-type mice to mammary, colon, ovarian, and skin tumors after exposure to carcinogens. PPAR-*γ* also enhances tumor formation in some genetic models of cancer. Significantly, troglitazone reduced the tumor incidence in wild-type but not heterozygote mice [107]. Chen et al. showed that the PPAR-*γ* ligand 15-Deoxy-Δ12,14-prostaglandin J2 (15dPGJ2), or ciglitazone, induces apoptosis in HT-29 by inhibiting NF-**κ**B activity, which upregulates various antiapoptotic genes, and by suppressing the expression of BCL-2, which protects cells against apoptosis [108]. In contrast, however, both troglitazone and rosiglitazone treatments increased the frequency and size of colon tumors in APC^min ^ mice, a clinically relevant model for both human familial adenomatous polyposis and sporadic colon cancer [109, 110]. These mice have a germ-line mutation of the APC gene resulting in deregulated *β*-catenin signaling and a very significantly increased frequency of small and large intestinal adenocarcinomas. Subsequently, however, generation of the APC^min ^ bigenic mouse with an intestinal-specific PPAR-*γ* deficiency demonstrated unequivocally that PPAR-*γ* suppresses tumor formation and suggested that thiazolidinedione has significant off-target effects in mice, especially in the APC^min ^ mouse colon cancer model [111]. These off-target effects of thiazolidinedione generally appear to have broad anticancerous properties; therefore, the findings in this model appear quite unusual.

 Clinical studies have not yet provided a conclusive answer to the question of whether PPAR-*γ* activity favors or inhibits cancer formation and progression, but their outcome has been largely disappointing and the clinical benefits were rather limited. The best results were obtained in three patients with liposarcoma, in which lineage-appropriate differentiation was induced [112]. At present, not enough evidence is available to establish with certainly whether PPAR-*γ* has pro- or antitumorigenic activities, and the field remains confusing. Some of this confusion, however, results from differences in experimental design. First, the differentiation state of the cells and tumors may affect the outcome. PPAR-*γ* activity is influenced by numerous other factors (cofactors, mutations in genes such as APC, and differentiation status). A final point is that the concentration of PPAR-*γ* agonist used is important. In breast cancer cell lines, for instance, low concentrations of PPAR-*γ* agonists induce cell proliferation; whereas higher concentrations of the same agonists correlate with cell cycle arrest and apoptosis [113]. A further point worth stressing is that troglitazone, a compound with significant antioxidant properties, is reported to be associated with the largest number of antitumorigenic effects.

 In contrast to normal cells and tissue, irradiation induces a progressive but significant tendency to overexpress PPAR-*γ* in HT-29 at one day after irradiation. Surprisingly, troglitazone treatment significantly reduced irradiation-induced PPAR-*γ* expression (personal communication) and may thus contribute to the lack of efficacy of its anticancer properties. At this time no information is available about any hypothetical deregulation of *β*-catenin signaling directly due to irradiation. Nonetheless, indirectly, irradiation is a nitric oxide (NO) producer, and NO induces *β*-catenin degradation and downregulates its transcriptional activity in colon cancer cells, thus revealing a so-far-unidentified mechanism of beta-catenin regulation [114]. The actions of these receptors may be attenuated in malignancies by genetic, cytogenetic, and environmental molecular mechanisms, which may compromise ligand generation. This understanding may have important implications for the necessary molecular diagnosis required to target PPAR*γ* therapies most effectively.

## 7. Conclusion

The development of gastrointestinal dysfunction after pelvic radiotherapy depends on a complex pathological process. These gastrointestinal symptoms affect the quality of life, are substantially more common than generally recognized and are frequently inadequately managed. They develop because radiation can induce changes in one or more specific physiological functions in widely separated parts of the gastrointestinal tract that lie in the path of the radiotherapy beam. The increasing weight of evidence suggesting a relation between acute and late effects may encourage clinicians to look at methods for decreasing tissue toxicity and thus improving the patient's quality of life. But decisions about the treatment options for reducing radiotherapy toxicity must be weighed after a careful benefit-risk analysis that depends on the target organ. Short-term preconditioning strategies with PPAR agonists can be protective in several animal models. Thus PPARs may provide a new strategy for intestinal injury prevention in radiation toxicity as in IBD. The tissue level of PPARs appears to be very radiosensitive. Accordingly some additional experiments may be needed to establish the mode of action of PPAR agonists in radiation protection and the stage at which PPARs can influence normal tissue self-renewal. A patient's ability to respond to appropriate therapy has a great impact on the actual toxicity of the treatment. However, as mentioned previously, it remains unclear whether PPARs act as oncogenes or as tumor suppressors. Further studies are needed to confirm this effect, especially in the absence of any effect on tumor control.
